# A longitudinal cohort study of rural adolescent vs adult South African mothers and their children from birth to 24 months

**DOI:** 10.1186/s12884-018-2164-8

**Published:** 2019-01-11

**Authors:** Karl le Roux, Joan Christodoulou, Linnea Stansert-Katzen, Elaine Dippenaar, Christina Laurenzi, Ingrid M. le Roux, Mark Tomlinson, Mary Jane Rotheram-Borus

**Affiliations:** 1 Walter Sisulu University Family Medicine Department, Zithulele Hospital, Mqanduli District, 5080 South Africa; 2 University of California, Los Angeles, USA; 3 Department of Psychology, Stellenbosch University, Private Bag X1, Matieland, 7602 South Africa

**Keywords:** Adolescent parenthood, Rural motherhood, Rural African children

## Abstract

**Background:**

Adolescent motherhood has been repeatedly linked to poor child outcomes in high income countries and urban areas in low- and middle-income countries. We examine the structural, personal, and caretaking challenges of adolescent mothers and their children in rural South Africa compared to adult mothers over the first 24 months post-birth.

**Methods:**

A cohort of sequential births (*n* = 470/493) in the rural OR Tambo District was recruited and reassessed at 3, 6, 9, 12, and at 24 months post-birth, with a retention rate above 84% at all timepoints. Maternal and child outcomes were examined over time using multiple linear and logistic regressions.

**Results:**

Adolescent mothers reflect 17% of births (*n* = 76/458). Adolescent mothers were more likely to have water in their households, but less likely to live with a partner and to be seropositive for HIV than adult mothers. Risks posed by mental health symptoms, alcohol, and partner violence were similar. Adolescents exclusively breastfed for shorter time and it took longer for them to secure a child grant compared to adult mothers. Although obtaining immunizations was similar, growth was significantly slower for infants of adolescent mothers compared to adult mothers over time.

**Conclusions:**

In rural South Africa, almost one in five pregnant women is an adolescent. Caretaking tasks influencing child growth, especially breastfeeding and securing the child grant appear as the greatest problems for adolescent compared to adult mothers.

## Background

Childbirth is the leading cause of death among young adolescent women aged 15–19 years old globally [1]. Each year, there are about 23 million births (12% of all births) to adolescents under 20 years of age. Newborns of adolescent mothers are twice as likely to suffer stillbirth and neonatal death and are more likely to be born with low birth weight, when compared to the babies of mothers who are 20–29 years old [2]. Adolescent parenthood is a significant global social and health problem [1] and is most common in sub-Saharan Africa (229/1000), compared to other regions (47/1000 globally) [2]. It is also up to three times more likely in rural areas compared to urban areas globally [1]. Yet, there are few data from rural Africa, the continent with the highest overall rate of adolescent motherhood. The current study aims to examine the rate of adolescent pregnancies in a deeply rural area of South Africa (SA), as well as to document the structural, personal risk histories, and caretaking of adolescent compared to adult mothers and compare their children’s development over the first 24 months post-birth.

Adolescent mothers often have less structural resources: they are often less educated and lack financial independence than adult mothers in both high (HIC) and low- and middle-income countries (LMIC) [3]. In SA, poverty is widespread and access to healthcare is limited for all mothers, especially in the deeply rural regions of the Eastern Cape. It may be that adolescents are more dependent on their families than young women may be in urban settings with greater resources [4].

Pregnant adolescents are likely to be those who have personal histories of risk – mental health or to make poor choices in their partners. Adolescent motherhood has been linked to mental health problems in HIC [5–7]. However, most adolescents live in LMIC, yet available prevalence data for these countries is limited [7]. Young age and poverty are also consistently associated with intimate partner violence (IPV) globally [8]. In African and Latin American countries, IPV is the highest during pregnancy when compared to nineteen other countries in Europe and Asia [9]. Mothers experiencing IPV during and after pregnancy are at risk for many physical and emotional adverse effects including physical injuries, HIV infection, unwanted pregnancies, and depression [10]. Further, children of mothers who experience IPV or depression can suffer growth delays as well as emotional and cognitive difficulties [11]. Therefore, we examine the relationship of personal risk histories between adolescent and adult mothers in rural South Africa in this paper. Considering the physical, social, emotional, and financial difficulties associated with pregnancy and child rearing in adolescence, a greater number of negative maternal risks are predicted among adolescent versus adult mothers.

Research suggests that poverty linked with young age contributes to insufficient perinatal care which can place the mother and child at risk for many health and developmental problems [12–15]. Many pregnant schoolgirls do not remain in school leading to a cycle of lifelong unemployment and poverty. These structural challenges reverberate to influence adolescent mothers’ caretaking. For example, those who return to school will often stop breastfeeding their babies which contributes to the low rates of exclusive breastfeeding (8% at 6 months) [16]. Adolescent mothers are predicted to have more difficulty in caretaking tasks such as breastfeeding and obtaining immunizations when compared to adult mothers. In this paper, we aim to examine the rate of adolescent pregnancy among a population cohort in the Eastern Cape of SA, an area typical of rural regions throughout Sub-Saharan Africa. We also aim to assess possible risks (e.g., HIV status, IPV, depressed mood) and protective factors (e.g., structural resources such as formal housing and household access to electricity and water, breastfeeding, immunizations) of adolescent and adult motherhood over the first 24 months of life.

## Methods

This study was conducted with approval of the Health Research Ethics Committee of Stellenbosch University (N12/08/046) and the Institutional Review Board at the University of California, Los Angeles (IRB#16–001362).

### Setting

Zithulele Hospital in the King Sabata Dalindyebo sub-district of the OR Tambo District of the Eastern Cape of SA. It is a 146-bed district hospital and has a catchment area with a population of approximately 130,000 people. It is situated in one of the two poorest municipalities in SA [17], in the former Transkei homeland, a part of SA which was systematically neglected under Apartheid.

Between January to April 2013, a cohort of consecutive of mothers giving birth at Zithulele Hospital and in the area covered by its 10 closest clinics were approached to participate in the study. This included mothers who delivered at home (10%) or on the way to a health facility (3%), and who sought follow-up clinic care for their child. Mothers who travelled to the hospital from outside this catchment area to give birth at the hospital were excluded from the sample. Voluntary informed consent was obtained (470/493 live births, 4.6% refusal rate), and, in the case where the mother was less than 18 years old, consent was also obtained from the parents/guardians of the adolescent mother. Mothers were approached while still in the hospital and typically interviewed in the first few days following birth. Women giving birth at clinics were assessed within the first 2 weeks of birth; those giving birth at home were approached at the first post-natal clinic visit.

Women visit clinics as soon as possible after giving birth where they secure a government Road to Health Card (RtHC). Not only is the RtHC a type of health passport and, therefore, an important health record, it also serves as proof of birth and is used to apply for a birth certificate, which is then used to apply for a child support grants (R250 per month in 2013) from the South African Social Security Agency (SASSA). We are therefore confident that that most mothers giving birth at home in our area were recruited.

Women were reassessed at five points in the next 24 months with high follow-up rates: 84.8% at 3, 92.1% at 6, 88.3% at 9, 91.3% at 12, and 88.0% at 24 months post-birth.

### Assessments

Local, isiXhosa-speaking women were recruited and trained as field workers over a 6-week period focusing on interview techniques, the ethics of research, confidentiality and the use of mobile phones as a data collection tool. Field workers collected data on mobile phones, which were pre-programmed with assessment questions by the Mobenzi mobile phone team (https://www.mobenzi.com/). The quality of the data entered into the Mobenzi Researcher Platform was checked on a weekly basis.

The following measures were completed at each assessment point.

### Maternal measures

Structural factors were identified as maternal age, highest education level obtained and whether they were currently in school, living with their partner or their partner’s family, household income, and having water on the premises (water tank), electricity, and the number of people living in the household.

#### Maternal health

Mothers self-reported whether they were primipara and how many antenatal visits they attended. This information was verified on the mothers’ antenatal cards when possible. HIV status was self-reported by mothers at each assessment. Maternal serostatus was confirmed using their maternity delivery record (available for all mothers who had clinic or hospital births) and/or on the child’s government-issued Road to Heath Cards (RtHC) (available for all children). **Maternal depression** was assessed using the Edinburgh Postnatal Depression Scale (EPDS) [18, 19]. We report both the mean scale score and identify mothers whose response indicate probable depressive disorder (> 13 to indicate depressed mood) [20].

**Intimate partner violence (IPV)** was self-reported at the baseline interview and the 12-month assessment with four items adapted from Jewkes and colleagues [21, 22]. Mothers were asked four items referring to the past 12 months: if they were slapped or had anything thrown at them; were pushed or shoved; were punched with a fist or another object; or were attacked or threatened with a weapon by their partner.

### Child measures

#### Received the child support grant (CSG)

Whether or not the mother was receiving the South African CSG was recorded at each assessment post-birth.

#### Breastfeeding

Mothers self-reported what they had fed their children in the past 24 h and the past week. Breastfeeding habits were specifically enquired about.

**Number of up-to-date immunizations** were recorded at each assessment based on the child’s RtHC and coded as complete (1) or incomplete (0).

**Low birth weight** was recorded for those infants < 2500 g (1) or ≥ 2500 g (0).

**Growth** was measured by field workers certified in using electronic scales and measuring mats. Children’s weight and height measures were then converted to z-scores based on the World Health Organization’s (WHO) age-adjusted norms (http://www.who.int/childgrowth/standards/en/) for: height-for-age (HAZ), weight-for-age (WAZ), and weight-for-length/height (WHZ). A Z-score below -2SD was considered a serious health deficit: as being stunted (<-2SD for HAZ), underweight for age (<− 2 for WAZ) or acute malnutrition (<− 2 for WHZ) [23].

#### Development

The gross motor developmental milestones of the WHO for children at 6 (WHO1), 9 (WHO3), 12 (WHO1–5) and 24 months (WHO1–6) were administered [24, 25]. These include six milestones that are fundamental to acquiring self-sufficient locomotion: sitting without support, hands-and-knees crawling, standing with assistance, walking with assistance, standing alone, and walking alone [26].

### Analysis

The primary analysis compared adolescent (< 18 years old) and adult mothers using mixed effects regression models, with adult mothers as the reference group. Logistic regression models were used for binary outcomes. Ten of the 470 mothers gave birth to twins, these mothers and their children were excluded in the current analysis. Two HIV-positive children were omitted from the analyses due to death and a coding error. All models were adjusted for repeated measures, where appropriate, and a random participant effect to control for the longitudinal nature of the assessments was used. The regression models were carried out using IBM SPSS Statistics (Version 20, Armonk, NY: IBM Corp).

## Results

Table 1 summarizes the characteristics of the adolescent and adult mothers at the point of recruitment. Adolescents were 17% of mothers (76/458). Over 24 months post-birth, 22 of the children whose mothers were recruited to the study died; five of these had adolescent mothers. The baseline assessment occurred within 2 weeks of birth for almost all mothers (81%) and within 1 month for all but one mother.

**Table 1:** Differences in the baseline characteristics between adolescent mothers (*n* = 76) and adult mothers (*n* = 382) assessed post-birth

	Adolescent Mothers *n* = 76 M (SD)/ % (n)	Adult Mothers *n* = 382 M (SD)/ % (n)	All Mothers *n* = 458 M (SD)/ % (n)
Structural Factors			
Mean age (SD)	16.18 (0.91)	26.49 (6.59)	24.78 (7.15) ***
Mean highest education level (SD)	8.21 (1.48)	8.68 (2.54)	8.60 (2.40)
Live with father or family	8% (6)	38% (146)	33% (152) ***
Monthly household income> 2000 Rand	44% (30/68)	49% (179/369)	46% (209/437)
Water tank on site	25% (19)	15% (56)	16% (75) *
Electricity	15% (11)	16% (60)	16% (71)
Number of people in household	6.08 (2.75)	5.86 (3.00)	5.89 (2.96)
Health			
Primipara	96% (73)	29% (111)	40% (184) ***
≥ Four antenatal appointments	41% (12/29)	48% (125/261)	47% (137/290)
HIV Seropositive	6%	33% (127)	29% (131) ***
EPDS > 13	11% (8)	17% (65)	16% (73)
Intimate Partner Violence (IPV)			
12 months prior to pregnancy	11% (8)	11% (43)	11% (51)
During pregnancy	22% (17)	22% (83)	22% (100)

Note. * p < .05; ** p < .01; *** p < .001

On average, all mothers received an eighth-grade education. More adult mothers lived with the father of their child or with their in-laws, compared to adolescent mothers (38% vs. 8%). About half of all households had monthly incomes under ZAR 2000 (about 144 USD). Only about 16% of households had a water tank on the premises; however, adolescent mothers were significantly more likely to have water on their household’s premises compared to adult mothers (25% vs. 15%). Only about 16% of households had any electricity in their households, with each household having eight members on average.

Almost all adolescent mothers were primaparous (95%); three adolescent mothers were pregnant for the second time and one was on her third pregnancy. On average, half of adolescent and adult mothers had the recommended minimum of four or more antenatal visits. Adolescents were significantly less likely to be HIV infected than adult mothers (6% vs. 33%). About 41% of mothers over 24 years of age were HIV seropositive; a rate almost double that of 18- to 23-year olds (24%) and more than five times that of adolescents (6%).

### Maternal outcomes

Depressive symptoms and the rate of depressed mood (EPDS > 13) were also similar at the time of birth between adolescent (*M* = 7.33, *SD* = 5.19; 11%) and adult mothers (*M* = 8.01, *SD* = 5.62; 17%). Compared to depression reported at birth, both adolescent and adult mothers reported fewer depressive symptoms over time. Depression was significantly lower at 3 months (*Estimate =* − 0.61, *Std Error* = 0.30, *p* = .04), 6 months (*Estimate =* − 1.21, *Std Error* = 0.35, *p* = .001), 9 months (*Estimate =* − 1.34, *Std Error* = 0.33, *p* < .001), 12 months (*Estimate =* − 1.08, *Std Error* = 0.36, *p* = .003), and 24 months post-birth (*Estimate =* − 2.64, *Std Error* = 0.36, *p* < .001).

Both adolescents and adults experienced IPV at the same rate with one in five women having been beaten in the past 12 months before learning about pregnancy. This rate increased significantly once the pregnancy was recognized for both adults and adolescents (11% vs. 22%), *B* = 0.84, *Std. Error* = .13, *p* < .001.

### Child caretaking & developmental outcomes

Table 2 summarizes caretaking and child outcomes over time for children with adolescent mothers and adult mothers. At birth, almost all mothers reported having applied for the child grant (95%), however, it took adolescents significantly longer to secure the grant compared to adult mothers, *B* = −.24, *Std. Error* = .48, *p* < .001. By 24 months of age, children were similarly receiving the child grant whether they had an adolescent or an adult mother. Adolescent mothers were less likely to exclusively breastfeed for 3 or 6 months when compared to adult mothers, *B* = − 0.72, *Std. Error* = .34, *p* = .04. Complete and up-to-date immunizations were similar over time for adolescent and adult mothers. Adolescent mothers were more likely to have returned to school by 6 months post-birth than adult mothers (17% vs. 2%).

**Table 2:** Child outcomes over time (assessments at 3, 6, 9, 12, and 24 months) for children grouped by adolescent mothers and adult mothers

		Follow-up Months					
		Birth	3 months	6 months	9 months	12 months	24 months
Secured Child Grant (CSG)	Adolescent	–	7% *	36% *	59% *	68% *	86%
	Adult	–	44% *	72% *	76% *	81% *	91%
Breastfed	Adolescent	–	13% *	12% *	–	–	–
Exclusively	Adult	–	23% *	22% *	–	–	–
Immunizations Current	Adolescent	–	42%	69%	83%	76%	71%
	Adult	–	50%	75%	84%	73%	74%
Mean HAZ (SD)	Adolescent	−0.3 (1.2) *	−0.1 (1.2) *	− 0.2 (1.4) *	− 0.2 (1.2) *	− 0.5 (1.2) *	− 0.4 (1.1) *
	Adult	− 0.1 (1.1) *	0.3 (1.2) *	0.2 (1.3) *	0.1 (1.4) *	−0.1 (1.4) *	− 0.2 (1.3) *
Mean WAZ (SD)	Adolescent	−0.8 (0.9) *	−0.6 (1.1) *	− 0.1 (1.1) *	0.1 (1.1) *	− 0.1 (1.2) *	0.0 (1.1) *
	Adult	−0.5 (1.0) *	−0.0 (1.1) *	0.1 (1.2) *	0.2 (1.2) *	0.2 (1.3) *	0.2 (1.1) *
Mean HWAZ (SD)	Adolescent	−0.7 (1.3)	−0.4 (1.3)	− 0.1 (1.2)	0.2 (1.2)	0.2 (1.2)	0.2 (1.3)
	Adult	−0.6 (1.3)	−0.3 (1.2)	0.1 (1.2)	0.3 (1.2)	0.4 (1.2)	0.4 (1.2)
HAZ < 2 SD	Adolescent	10%	3%	7%	8%	6%	8%
Stunting	Adult	6%	3%	5%	7%	8%	7%
WAZ < 2 SD	Adolescent	12%	9%	5%	3%	3%	3%
Malnourished	Adult	8%	3%	4%	3%	4%	3%
WHZ < 2 SD	Adolescent	20%	15%	6%	3%	5%	5%
	Adult	17%	8%	4%	2%	3%	3%
WHO Milestones Completed	Adolescent	–	–	80%	94%	76%	97%
	Adult	–	–	91%	94%	77%	99%

Note * *p* < .05

**Fig. 1:**
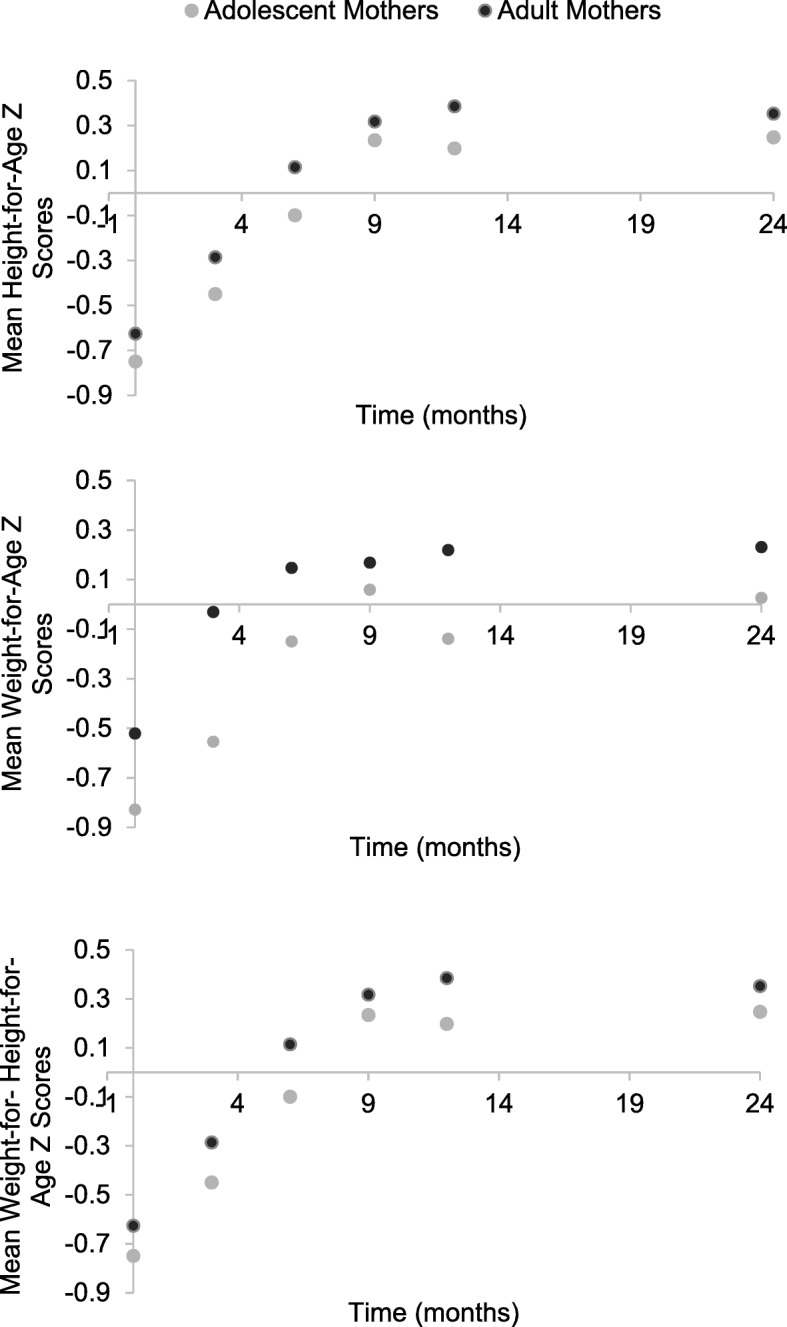
The effect of being an adolescent mother on height-for-age z-scores (HAZ), weight-for-age z-scores (WAZ), and weight-for-height-for-age (WHZ)

At birth, the prevalence of infants with low birth weights was similar in children of adolescent and adult mothers (10%). However, children of adolescent mothers tended to be significantly shorter and weighed less than children of adult mothers across their first 24 months of life, *Estimate* = −.26, *Std. Error* = 0.11, *p* = .02; *Estimate* = −.32, *Std. Error* = 0.12, *p* = .01, respectively. WAZ and WHZ increased significantly for children of adolescent and adult mothers from birth to 3 (*Estimate* = .49, *Std. Error* = .05, *p* < .001; *Estimate* = .34, *Std. Error* = .09, *p* < .001, respectively) 6 (*Estimate* = .70, *Std. Error* = .06, *p* < .001; *Estimate* = .76, *Std. Error* = .08, *p* < .001, respectively) 9 (*Estimate* = .75, *Std. Error* = .06, *p* < .001; *Estimate* = .99, *Std. Error* = .08, *p* < .001, respectively) 12 (*Estimate* = .73, *Std. Error* = .07, *p* < .001; *Estimate* = 1.00, *Std. Error* = .08, *p* < .001, respectively) and 24 (*Estimate* = .76, *Std. Error* = .06, *p* < .001; *Estimate* = 1.00, *Std. Error* = .08, *p* < .001, respectively) months post-birth. However, HAZ increased significantly for children of adolescent and adult mothers from birth to only 3 (*Estimate* = .39, *Std. Error* = 0.07, *p* < .001) and 6 (*Estimate* = .29, *Std. Error* = 0.07, *p* < .001) months post-birth. HAZ did not change significantly from birth to 6, 8, 12, or 24 months post-birth *p* > .05 (See Fig. 1). The rate of stunting, malnourishment, and wasting among children of adolescent and adult mothers was similar at birth and over time. At 24 months post-birth, 7% of children were stunted, 3% were malnourished, and 3% were wasted.

## Discussion

In rural SA, almost one in five pregnant women is an adolescent. With about half of adolescent and adult mothers living in households with reported incomes under ZAR 2000 and limited support from their partners, obtaining the child grant becomes even more important to support mothers and their children. Adolescent mothers experience significant lags in securing child grants compared to adult mothers. However, the reason for the delays in obtaining these child grants is unclear. This puts an already at-risk group in socio-economic jeopardy. Delays in obtaining these funds may be particularly difficult for adolescent mothers who have limited means of income, potentially resulting in elevated risks for malnutrition and stunting for their children.

Breastfeeding serves as a protective factor for both adolescent and adult mothers. Overall, the rate of exclusively breastfeeding during the first 6 months of life is higher than the national rate of 8%; however, adolescent mothers are significantly less likely to exclusively breastfeed than adult mothers [10]. This can be partially explained by adolescent mothers returning to school after the birth of their child. By 6 months-post birth, almost one in five adolescent mothers return to school. Prior research suggests that adolescent mothers living in rural areas are more likely to return to school than their peers living in urban areas [27]. However, the rate of matriculation among adolescent mothers is still low. Pregnancy is one of the most common reasons for dropping out of school among young women and returning to school may be difficult not only because of the increased demands of caretaking but because of teacher opposition to having pregnant or young mothers in their classrooms. Future work needs to examine the social structural factors associated with adolescent mothers’ access to education after the birth of their child.

As predicted, adolescent mothers face increased difficulties in caretaking. Although the percentage of low birth weight infants and stunted children are similar for adolescent and adult mothers, children of adolescent mothers are significantly shorter and weighed less on average than those of adult mothers over time. Lags in early physical growth are associated with negative health, social, and cognitive outcomes across the lifespan [28]. The reduced rate of exclusive breastfeeding among adolescent mothers may explain some of these deficits in child growth.

Acquiring HIV, IPV, and, depression are also a significant risk factor for young women who fall pregnant in SA. The pathways to HIV infection among adolescents include unprotected and intergeneration sex [29], as well as acquiring HIV during their own mother’s pregnancies, deliveries, or lactation [30–32]. Adolescent women in SA are three times more likely to be HIV positive, when compared to men their age (15.5% vs. 4.8%) [33]. Yet, HIV infection increases with age in Africa, so the HIV rates are likely lower among adolescent, compared to adult mothers. Consistent with previous findings in peri-urban regions in SA [34], one in five adolescent and adult mothers report interpersonal violence. IPV is a power issue; being young consistently confers less power within a relationship. The increasing rates of IPV likely reflect the increased and sustained stress associated with child bearing, especially for young, first-time mothers.

## Limitations

Although children’s growth and developmental milestones are measured by the trained interviewers at each assessment, most of the maternal measures are based on maternal self-reports. The HIV status of most mothers is self-reported at each assessment and on their maternity delivery record and their children’s RtHC, which are used to confirm their serostatus. However, no laboratory tests are available to confirm maternal HIV status or to test children’s immune systems.

## Conclusions

In conclusion, there is a high rate of adolescent pregnancies in the rural Eastern Cape of SA. The impact on adolescent mothers in terms of their caretaking is significant and their babies are also shorter and weigh less on average, than babies of adult mothers. This situation underlines the importance of supporting adolescent mothers, especially those reporting difficulties in acquiring the CSG. Also, ensuring that local officials support all mothers 16 years and over to apply for the CSG immediately may assist in providing essential economic support for adolescent mothers and their children.

## Abbreviations

AFR: Adolescent fertility rate
EPDS: Edinburgh Postnatal Depression Scale
HAZ: Height-for-age
HIC: High
IPV: Intimate partner violence
LMIC: Low and Middle Income Countries
MLH: Mothers living with HIV
MWOH: Mothers living without HIV
RtHC: Government Road to Health Card
SA: South Africa
SASSA: South African Social Security Agency
WAZ: Weight-for-age
WHO: World Health Organization
WHZ: Weight-for-length/height
